# Prognostic value of thrombin generation parameters in hospitalized COVID-19 patients

**DOI:** 10.1038/s41598-021-85906-y

**Published:** 2021-04-08

**Authors:** María Eugenia de la Morena-Barrio, Carlos Bravo-Pérez, Antonia Miñano, Belén de la Morena-Barrio, María Piedad Fernandez-Perez, Enrique Bernal, José Miguel Gómez-Verdu, María Teresa Herranz, Vicente Vicente, Javier Corral, María Luisa Lozano

**Affiliations:** 1 Servicio de Hematología y Oncología Médica, Hospital General Universitario Morales Meseguer, Centro Regional de Hemodonación, Universidad de Murcia, IMIB-Arrixaca, CIBERER, Avda. Marqués de Los Vélez, s/n, 30008 Murcia, Spain; 2 Servicio de Enfermedades Infecciosas, Hospital Reina Sofía, Murcia, Spain; 3 Servicio de Medicina Interna, Hospital Universitario Morales Meseguer, Murcia, Spain

**Keywords:** Cardiovascular diseases, Haematological diseases, Infectious diseases, Respiratory tract diseases

## Abstract

SARS-CoV-2 infection increases the risk of thrombosis by different mechanisms not fully characterized. Although still debated, an increase in D-dimer has been proposed as a first-line hemostasis test associated with thromboembolic risk and unfavorable prognosis. We aim to systematically and comprehensively evaluate the association between thrombin generation parameters and the inflammatory and hypercoagulable state, as well as their prognostic value in COVID-19 patients. A total of 127 hospitalized patients with confirmed COVID-19, 24 hospitalized patients with SARS-CoV-2-negative pneumonia and 12 healthy subjects were included. Clinical characteristics, thrombin generation triggered by tissue factor with and without soluble thrombomodulin, and also by silica, as well as other biochemical parameters were assessed. Despite the frequent use of heparin, COVID-19 patients had similar thrombin generation to healthy controls. In COVID-19 patients, the thrombin generation lag-time positively correlated with markers of cell lysis (LDH), inflammation (CRP, IL-6) and coagulation (D-dimer), while the endogenous thrombin potential (ETP) inversely correlated with D-dimer and LDH, and positively correlated with fibrinogen levels. Patients with more prolonged lag-time and decreased ETP had higher peak ISTH-DIC scores, and had more severe disease (vascular events and death). The ROC curve and Kaplan Meier estimate indicated that the D-dimer/ETP ratio was associated with in-hospital mortality (HR 2.5; *p* = 0.006), and with the occurrence of major adverse events (composite end-point of vascular events and death) (HR 2.38; *p* = 0.004). The thrombin generation ETP and lag-time variables correlate with thromboinflammatory markers, and the D-dimer/ETP ratio can predict major adverse events in COVID-19.

## Introduction

The clinical manifestations of SARS-CoV-2 infection are highly heterogeneous, from asymptomatic disease to devastating outcomes. The most characteristic complication is acute respiratory distress syndrome (ARDS), but thrombotic complications are also frequent, especially in patients admitted to the intensive care unit (ICU)^1–8^. Thrombosis may be justified by excessive inflammation, hypoxia, immobilization, and microvascular injury^9–14^. Despite the strides that have been made in the last months in understanding the mechanisms underlying COVID-19 associated coagulopathy, efforts are still necessary to decipher its complexity. The hemostatic profile of COVID-19 infection is different from sepsis coagulopathy or disseminated intravascular coagulation (DIC)^15–20^. Endothelial dysfunction and platelet activation may support thrombin generation. The activation of the complement system pathways may contribute to the production of cytokines and chemokines, and also phenomena of hypofibrinolysis could ultimately lead to immunothrombosis^21^. With limited solid data available, it is crucial to identify biomarkers that may help predict poor outcomes, including thrombotic events. In addition to D-dimer, most attempts have been mainly focused on the detection of new thromboinflammatory biomarkers, but studies analyzing global hemostatic methods are rare. In this disease, the correlation of thrombin generation parameters with biomarkers of immunothrombosis or with clinical outcomes has not been evaluated, which could provide some additional useful information not only in our understanding of the physiopathology of the disease but could also help for stratification and managed care of COVID-19. The aim of this study was to systematically investigate the potential association of thrombin generation with other biochemical variables, and to assess the prognostic value of this assay in patients with COVID-19.

## Materials and methods

### Patients

A total of 142 consecutive patients with COVID-19 requiring hospital admission at Morales Meseguer and Reina Sofia University Hospitals (Murcia, Spain) from March to April 2020 were eligible for this cross-sectional study. In all patients, SARS-CoV-2 infection was assessed by quantitative real-time reverse-transcriptase polymerase chain reaction (qRT-PCR) using the diagnostic criteria defined in the World Health Organization (WHO) interim guidance^22^. Exclusion criteria included chronic oral anticoagulation at the time of blood sampling, patients younger than 18 years of age, or those with missing data or who were transferred from other designated hospitals. Final follow-up date July 14, 2020.

As comparative groups, patients requiring admission at the same period with SARS-CoV-2-negative pneumonia were also enrolled. In these cases, COVID-19 diagnosis was ruled out by repetitively negative SARS-CoV-2 qRT-PCR and serologic testing. Samples from 12 healthy individuals and a pool of plasma from 100 healthy subjects were also analyzed for thrombin generation.

### Ethics approval and consent to participate

All included subjects gave their informed consent to enter the study, which was approved by the Ethics Committee of Morales Meseguer Hospital, and performed in accordance with the 1964 Declaration of Helsinki and their later amendments.

### Sample collection for thrombin generation analysis

Samples were collected during hospitalization in vacuum tubes containing 0.109 M sodium citrate. Platelet-poor plasma (PPP) was obtained within 4 h after extraction by two cycles of centrifugation (2000 g × 15 min) at 20 °C and then stored at -80 °C.

### Data collection

Three investigators (MTH, JMGV, and EB) independently extracted data including demographics, comorbidities, laboratory studies and outcomes, using a standardized data collection form for analysis. Such data were checked independently by three investigators (CBP, MPFP and MEMB) to verify accuracy. The clinical information comprised patient demographics, medical comorbidities, prior thrombotic history, chronic anticoagulation, length of hospital stay, CURB-65 score at admission, development of ARDS, ICU admission, sequential organ failure assessment (SOFA) score, vascular (thrombotic/ hemorrhagic) events, antithrombotic therapy while hospitalization, and death.

Venous and arterial thrombotic events were confirmed by radiologic methods (Doppler-Ultrasonography [US] and Computed Tomography [CT]). Bleeding was graded according to World Health Organization (WHO) scale. Grade ≥ 3 bleeding episodes, requiring transfusion, were confirmed by angiographic procedures. Routine laboratory parameters: lactate dehydrogenase (LDH), C reactive protein (CRP), interleukin 6 (IL-6), complete blood cell counts, basic coagulation tests (prothrombin time [PT], activated partial thromboplastin time [aPTT], fibrinogen and D-dimer), were recorded at admission. They were then assessed at average intervals of 48 h, and their peak levels during follow-up were also documented. COVID-19 associated coagulopathy was graded according to DIC score of the International Society on Thrombosis and Haemostasis (ISTH)^23^. Admission and peak ISTH-DIC scores were calculated. Prescribed antithrombotic therapy with low molecular weight heparin (LMWH), specifically enoxaparin, was classified as standard prophylaxis (40 mg QD), high-risk prophylaxis (1 mg/Kg QD), and therapeutic regimens (1 mg/Kg BD).

### Thrombin generation assay

The thrombin generation assay (TGA) was performed using the calibrated automated thrombogram (CAT) method^24^. All samples were evaluated in the same equipment during three working days using the same reagent batch under the following conditions: samples were thawed at 37 °C and mixed with two different activators: PPP reagent (5 pM tissue factor [TF]; 4 µM phospholipids) (Stago Diagnostics) or silica (dilution 1/16 from Synthasyl reagent; Werfen), in a 96-well plate (Immulon, 2HB clear U-bottom; ThermoFisher Scientific). All samples were run in duplicate. Coagulation was triggered with calcium chloride in buffer containing the fluorogenic substrate (FluCa-kit reagent, Stago Diagnostics). For each individual plasma sample, we used a thrombin calibrator (Stago Diagnostics) to correct for differences in sample color, inner filter fluorescence, and substrate consumption. Fluorescence was recorded for 60 min in a Fluoroskan Ascent microplate fluorimeter (Thermolab Systems, Helsinki, Finland), and the data were then analyzed using Thrombinoscope software (version 5.0.0.742, Stago Diagnostics). The endogenous thrombin potential (ETP), the thrombin peak, the time to the thrombin peak, the velocity index and the lag-time were recorded for each assay. To assess the impact of the protein C anticoagulant pathway on the TGA, experiments activated with PPP reagent were also conducted in the presence of recombinant human soluble thrombomodulin (sTM) (American Diagnostica) at a final concentration of 7.5 nM. An index “R” described by Perrin et al^25^, was calculated for the TGA parameters, i.e. the value in the presence of sTM divided by the value in the absence of sTM; the closer the ratio is to 1, the weaker the response to the protein C system.

This assay was done in the baseline sample for both COVID-19 and the non-COVID-19 pneumonia groups.

### Statistical analysis

Descriptive analysis of qualitative variables included percentages. Pearson’s Chi-Squared test and Fisher’s Exact test were used for comparison of proportions or ordinal variables. Kolmogorov–Smirnov and Shapiro–Wilk tests were used for testing normality of continuous variables. Normally distributed continuous variables were presented as means ± standard deviations (SD), whereas non-normally distributed variables were presented as median and interquartile ranges (IQR). Student’s-T (parametric) or Mann–Whitney-U tests were used for comparison of two means and analysis of variance (ANOVA, parametric) or Kruskal–Wallis (non-parametric) tests were used for comparison of more than two means. Apart from *p* values, 95% Confidence Intervals (95%CI) were also calculated.

Association between thrombin generation and other continuous laboratory variables was assessed by Pearson’s (parametric) or Spearman’s Rho (non-parametric) correlation tests.

The predictive impact of thrombin generation and other laboratory parameters on the incidence of clinical events (hemorrhage, thrombosis, ARDS, ICU admission and death) was assessed by Cox regression analysis. Survival analysis was assessed using the Kaplan–Meier method and log-rank tests. Receiver-Operating-Characteristic (ROC) curve analysis was also performed, and optimal cut-off values were selected by means of maximizing sensitivity and specificity. Logarithmic and square root transformations of continuous variables were performed to make the data more closely meet the assumptions of statistical procedures to be applied, or to improve their interpretability.

Statistical analysis was performed with the use of Excel (Microsoft), GraphPad Prism (GraphPad Software), IBM SPSS Statistics 21 (IBM SPSS Software) and STATA v.14 (StataCorp LLC)^26^.

## Results

### Patients’ characteristics

A total of 127 out of 142 hospitalized COVID-19 patients were eventually included in the study. Fifteen patients were excluded: six due to oral anticoagulation uptake at the time of blood sampling and nine because of missing data or transfer from other hospitals.

The basic characteristics for all included participants are shown in Table 1. COVID-19 patients were older than those with SARS-CoV-2 negative pneumonia (median age 60 years [IQR, 47.5–72.3 years] vs. 37 years [IQR 29.5–31.7 years]), respectively; *p* < 0.001). The median time from admission to discharge was 10 days in COVID-19 patients, and 2 days in SARS-CoV-2 negative pneumonia patients (*p* = 0.002). COVID-19 patients had significantly higher rates of hypertension, diabetes, dyslipidemia, and chronic renal failure, whereas SARS-CoV-2 pneumonia patients had higher rates of history of smoking (*p* ≤ 0.05). The results also showed that 30.7%, and 26.0% of COVID-19 patients developed ARDS and required ICU admission, respectively, and that 7.9% of the COVID-19 cohort had fatal outcomes. Patients in the SARS-CoV-2-negative pneumonia group had much milder clinical manifestations.

**Table 1:** Demographic and clinical characteristics of patients with COVID-19 and SARS-CoV-2 negative pneumonia.

	COVID-19 (N = 127)	SARS-CoV-2-negative Pneumonia (N = 24)	*p*
Demographics			
Sex (females)	43.3% (N = 55)	54.2% (N = 13)	0.327
Age (years), median (IQR)	60 (47.5–72.3)	37 (29.5–31.8)	** < 0.001**
Comorbidities			
Hypertension	47.0% (N = 61)	12.5% (N = 3)	**0.002**
Diabetes mellitus	18.1% (N = 23)	0%	**0.016**
Dyslipidemia	36.2% (N = 46)	0%	** < 0.001**
Cardiovascular disease	14.2% (N = 18)	4.2% (N = 1)	0.135
Chronic lung disease	1.6% (N = 2)	8.3% (N = 2)	0.089
Smoking history	17.3% (N = 22)	37.5% (N = 9)	**0.049**
Chronic renal disease	18.9% (N = 24)	0%	**0.020**
Cancer	4.7% (N = 6)	0%	0.590
Immunosuppression	4.7% (N = 6)	0%	0.590
At-admission severity			
CURB-65 ≥ 2, % (95%CI)	30.7% (22.8–39.5%) (N = 39)	0% (0–14.2%)	**0.002**
Length of stay, median (IQR)	10 days (5–19 days)	2 days (1–3.8 days)	** < 0.001**
Evolution to critically-ill disease			
ARDS, % (95%CI)	30.7% (22.8–39.5%) (N = 33)	0% (0–14.2%)	**0.002**
ICU admission, % (95%CI)	26.0% (18.6–34.5%) (N = 39)	0% (0–14.2%)	**0.005**
SOFA score, median (IQR)	0 (0–2)	0 (0–1)	**0.020**
SOFA of ICU patients, median (IQR)	4 (0–7.5)	–	***–***
Deaths, % (95%CI)			
Overall	7.9% (3.8–14.0%) (N = 10)	0%	0.004*
ICU	18.2% (7.0–35.5%) (N = 6)	(0–14.2%)	
Non-ICU	4.3% (1.3–4.2%) (N = 4)		

Bold values denote statistical significance at the *p* < 0.05 level.

*ARDS* acute respiratory distress syndrome, *ICU* intensive care unit, *IQR* interquartile range, *SOFA score* Sequential organ failure assessment score.

*p value of Chi-Squared test for 3 categories (COVID-19 ICU and non-ICU and SARS-CoV-2-negative Pneumonia) and *p* value of 0.019 for deaths in ICU *vs.* non-ICU COVID-19 patients.

### Antithrombotic prophylaxis, thrombosis, and bleeding events in COVID-19 patients

Antithrombotic therapies, thrombotic events, and bleeding complications in patients with COVID-19 and with SARS-CoV-2 negative pneumonia are summarized in Table 2.

**Table 2:** Antithrombotic therapies and vascular events in COVID-19 patients and with SARS-CoV-2 negative pneumonia.

	COVID-19 (N = 127)	SARS-CoV-2 negative Pneumonia (N = 24)	*p*
**Antithrombotic therapy**			
None, %	22.0% (N = 28)	45.8% (N = 11)	**0.015**
Prophylactic LMWH, %	65.4% (N = 83)	54.2% (N = 13)	0.296
Intermediate/treatment dose LMWH, %	12.6% (N = 16)	0%	0.076
**Thrombotic events, % (95%CI)**			
Overall	5.5% (2.2–11.0%) (N = 7)	0% (0–14.2%)	0.004*
ICU	15.2% (5.1–35.9%) (N = 5)		
Non-ICU	2.1% (0.3% -7.5%) (N = 2)		
**Bleeding events, % (95%CI)**			
Overall	3.8% (1.2–8.6%) (N = 5)	0% (0–14.2%)	0.448*
ICU	6.1% (0.7–20.2%) (N = 2)		
Non-ICU	3.2% (0.7–9.0%) (N = 3)		

Bold values denote statistical significance at the *p* < 0.05 level.

*ICU* intensive care unit, *LMWH* low molecular weight heparin.

*p value of 0.013 for thrombotic events in ICU *vs.* non-ICU COVID-19 patients, and *p* value of 0.604 for bleeding events in ICU *vs.* non-ICU COVID-19 patients.

In the COVID-19 cohort, 7 patients experienced 8 thrombotic events. These included 5 venous episodes (4 pulmonary embolism [PE], 1 extensive superficial venous thrombosis), and 3 ischemic episodes (1 stroke, 1 acute coronary syndrome, and 1 acute peripheral arterial ischemia). Thus, the incidence of thrombosis in COVID-19 patients in our cohort was 5.5% (95%CI 2.2–11.0%); 3.9% (95%CI 1.3–8.9%) for radiographically-confirmed venous thromboembolism and 2.4% (95%CI 0.5–6.7%) for arterial thrombosis. Five out of these 7 patients (71.4%) had critically-ill COVID-19 and required ICU admission, rendering an incidence of thrombosis of 15.2% (95%CI 5.1–31.9%) in ICU patients. One of the patients developed two vascular events, a symptomatic PE followed 7 days later by acute myocardial infarction despite full-dose anticoagulation with heparin. Of note, thrombotic complications seemed to occur in spite of antithrombotic measures, since 85.8% of patients with thrombosis received pharmacological thromboprophylaxis, and 28.6% of them intermediate/therapeutic dose heparin.

Five bleeding events occurred in 5 COVID-19 patients: 2 retroperitoneal hematomas, 1 rectus sheath hematoma (these 3 episodes had a WHO Severity Grade ≥ 3), 1 upper gastrointestinal bleeding and 1 episode of hemoptysis. Therefore, the rate of bleeding in COVID-19 patients from our cohort was 3.8% (95%CI 1.2–8.6%) and 2.3% (95%CI 0.5–6.5%) for WHO grade ≥ 3 bleeding. Two out of these 5 patients (40.0%) had critically-ill COVID-19 and required ICU admission, resulting in an incidence of bleeding of 6.1% (95%CI 0.7–20.2%) in ICU patients. All cases received thromboprophylaxis with heparin. Notably, at the moment of bleeding, the 3 patients with Grade ≥ 3 internal hemorrhages were on intermediate/therapeutic-dose heparin.

No vascular events were recorded in patients with SARS-CoV-2-negative pneumonia.

### Laboratory data

For all patients, the baseline timepoint corresponded with the sample collected at a median of 2.0 days following admission (IQR: 1.0–7.0).

Baseline and peak levels of LDH and D-dimer were higher in COVID-19 patients than those in the SARS-CoV-2 negative pneumonia group. In terms of blood counts and other coagulation indexes, the COVID-19 group showed significantly lower trough platelet counts and PT compared with the SARS-CoV-2 negative pneumonia group. Thus, in the COVID-19 cohort, 11.0% of patients had thrombocytopenia (< 150 × 10^9^/L), 30.7% had prolonged PT (< 75%) and/or aPTT ratio (> 1.30) and 0.8% fibrinogen consumption (< 100 mg/dL). Meanwhile, baseline and peak ISTH-DIC score values were also significantly higher in COVID-19 patients than those in the SARS-CoV-2 negative pneumonia group (Table 3).

**Table 3:** Baseline and peak laboratory parameters in COVID-19 patients and in those with SARS-CoV-2 negative pneumonia. Data are shown as mean ± SD.

	Baseline		Peak value^a^		Normal range
	COVID-19	SARS-CoV-2-negative Pneumonia	COVID-19	SARS-CoV-2-negative Pneumonia	
**Inflammation markers**					
LDH, IU/L	505 ± 213	336 ± 65	668 ± 385	381 ± 77	208–378
*p*	** < 0.001**		** < 0.001**		
CRP, mg/dL	8.2 ± 8.3	7.2 ± 6.4	11.1 ± 9.8	7.5 ± 6.9	0–1.0
*p*	0.79		0.12		
IL-6, pg/mL	ND	ND	98.6 ± 304.7	16.0 ± 13.0	0–4.4
*p*	*–*		0.065		
**Routine hemostatic tests**					
Platelets, × 10^9^/L	216.2 ± 87.3	242.2 ± 89.0	194.9 ± 87.3	245.9 ± 89.1	150.0–450.0
*p*	0.11		**0.008**		
PT, %	91.8 ± 17.9	93.6 ± 12.6	79.8 ± 17.2	85.0 ± 13.3	75–100
*p*	0.68		**0.22**		
aPTT, ratio	1.06 ± 0.19	1.04 ± 0.12	ND	ND	0.80–1.30
*p*	0.79		***–***		
Fibrinogen, mg/dL	457 ± 189	482 ± 150	514 ± 180	508 ± 153	150.0–400.0
*p*	0.76		0.74		
D-dimer, mg/dL	1,308 ± 2,042	556 ± 464	4,162 ± 9,492	690 ± 489	0–500
*p*	**0.001**		** < 0.001**		
**ISTH-DIC score**					
Median, IQR	0 (0–1)	0 (0–0.25)	1 (0–2)	0 (0–1)	˂ 1
*p*	**0.024**		**0.001**		

Bold values denote statistical significance at the *p* < 0.05 level.

*aPTT* activated partial thromboplastin time, *CRP* C-reactive protein, *IL-6* interleukin-6, *LDH* lactate dehydrogenase, *PT* prothrombin time, *O.D.* optical density.

^a^Peak or minimum values of each laboratory parameter are shown, depending on its upward or downward tendency during COVID-19, respectively.

*P* values were based on Mann–Whitney-U test or T-test.

Notably, at admission, no patient met criteria of overt DIC (score ≥ 5), but in 51.9% of them, the profile was consistent with low-grade coagulopathy (scores 1–4). During follow-up, 64.7% cases showed low-grade coagulopathy, and 3.8% eventually met criteria of overt DIC. Both baseline and peak ISTH-DIC scores were higher in patients suffering from bleeding than those who did not (median baseline ISTH-DIC score: 2 [IQR: 1.25–2.75] for bleeders and 0 [IQR: 0–1] for non-bleeders, *p* = 0.005; median peak ISTH-DIC score: 3.5 [IQR: 1.5–4] for bleeders and 1 [IQR: 0–2] for non-bleeders, *p* = 0.016).

### Thrombin generation

The results of the thrombin generation parameters in COVID-19 and SARS-CoV-2 negative pneumonia evaluated in baseline samples, as well as the reference values of healthy subjects are shown in Table 4. Although 78.9% of COVID-19 patients (N = 105) were undergoing LMWH treatment, when thrombin generation was triggered either by TF or silica, COVID-19 patients had similar lag-time and ETP but higher peak than healthy controls. At 5 pM TF, SARS-CoV-2 negative pneumonia patients presented with a shorter lag-time, higher ETP and higher peak height compared to COVID-19 samples, most likely due to the lower proportion of patients receiving LMWH than COVID-19 patients (Table 4 and Fig. S1). In the study sample, when sTM was added, as an agent that converts thrombin into an anticoagulant capable of activating protein C, it could not significantly reduce the ETP in patients with pneumonia (COVID-19 and SARS-CoV-2 negative), compared with the effect expected and observed in samples from healthy subjects (Table 4).

**Table 4:** Baseline thrombin generation parameters of plasma samples from pneumonia patients with or without COVID-19 and healthy controls. Values are expressed as median [IQR].

TGA parameter	Activator	COVID-19 (N = 127)	SARS-CoV-2 negative (N = 24)	*p*	Healthy subjects (N = 12) & pool*	*p (vs COVID-19)*
Lag-time (min)	TF (5 pM)	3.83 [3.33–4.50]	3.33 [3.00–3.79]	**0.009**	3.36 [2.69–4.50]	0.284
	Silica (1/16)	3.00 [2.50–3.33]	3.02 [2.67–3.36]	0.527	3.33 [2.59–3.43]	0.502
ETP (nM/min)	TF (5 pM)	1263 [1101–1488]	1506 [1235–1747]	**0.005**	1169 [1092–1732]	0.376
	Silica (1/16)	1390 [1205–1610]	1556 [1304–1851]	**0.011**	1501 [1440–1732]	0.144
Peak (nM)	TF (5 pM)	224 [179–256]	237.3 [206–269]	**0.037**	119 [92–236]	**0.011**
	Silica (1/16)	191 [166–222]	190.0 [172–225]	0.696	171 [157–240]	0.392
Tt Peak (min)	TF (5 pM)	6.67 [6.00–7.67]	6.20 [5.33–6.86]	**0.030**	8.54 [5.36–11.67]	0.226
	Silica (1/16)	5.83 [5.17–6.50]	6.00 [5.40–6.70]	0.411	6.67 [5.60–6.85]	0.141
Vel Index (nM/min)	TF (5 pM)	79.3 [56.6–103.3]	91.8 [70.8–111.9]	0.139	23.1 [13.8–90.6]	**0.007**
	Silica (1/16)	67.0 [55.1–84.1]	64.5 [58.3–83.9]	0.800	49.7 [45.6–87.5]	0.147
Ratio ETP sTM/No	TF (5 pM)	0.86 [0.76–0.91]	0.86 [0.82–0.89]	0.706	0.63 [0.52–0.89]	**0.023**

Bold values denote statistical significance at the *p* < 0.05 level.

*Plasma pool of 100 blood donors.

*sTM* soluble thrombomodulin, *TF* Tissue factor, *TGA* thrombin generation assay, *Tt Peak* time to peak, *Vel Index* velocity index.

*P* values were based on Mann–Whitney-U test.

### Correlation of thrombin generation parameters with analytical data in COVID-19 patients

Analysis of the association between baseline TGA parameters and peak values of all other analytical data was performed. Peak values were dichotomized according to the median. As shown in Table 5, in COVID-19 patients higher LDH, CRP, IL-6, and D-dimer values were associated with longer lag-time triggered by TF. Additionally, increased peak LDH and D-dimer levels, but lower peak fibrinogen values were associated with lower ETP at 5 pM TF (Table 5).

**Table 5:** Association between baseline TGA parameters (lag-time and ETP) induced by TF (5 pM) and peak analytical data in COVID-19 patients.

Median (N)	LDH (IU/L)		CRP (mg/L)		IL-6 (pg/mL)		Fib (mg/dL)		D-D (mg/L)	
	< 564 (62)	> 564 (65)	< 8.6 (71)	> 8.6 (56)	< 13.8 (75)	> 13.8 (52)	< 446 (66)	> 446 (61)	< 1.24 (63)	> 1.24 (64)
**Lag-time (min)**										
Mean ± SD	3.7 ± 1.1	4.3 ± 1.2	3.9 ± 1.3	4.2 ± 0.9	3.8 ± 1.1	4.3 ± 1.1	4.1 ± 1.3	3.9 ± 0.9	3.7 ± 0.7	4.4 ± 1.3
p*	**0.022**		0.01		**0.004**		0.969		** < 0.001**	
Correlation Rho	0.247		0.0.218397		0.407		0.068			
p**	**0.007**		** < 0.0010.021**		** < 0.001**		0.461			
**ETP (nM/min)**										
Mean ± SD	1350 ± 320	1222 ± 270	1267 ± 330	1307 ± 260	1264 ± 300	1314 ± 300	1235 ± 340	1337 ± 240	1354 ± 280	1216 ± 300
p*	**0.016**		0.19		0.368		**0.022**		**0.019**	
Correlation Rho	-0.241		0.024		-0.046		0.300		-0.288	
p**	**0.009**		0.804		0.647		**0.001**		**0.001**	

Bold values denote statistical significance at the *p* < 0.05 level.

Peak values of LDH, CRP, IL-6, Fib and D-D are dichotomized according to the median.

*Mann–Whitney-U test and on **Spearman’s Rho correlations.

*Rho* Spearman’s Rho (range: − 1.00 to + 1.00), *ETP* Endogenous thrombin potential, *LDH* Lactate dehydrogenase, *CRP* C reactive protein, *IL* Interleukin, *O.D.* optical density, *Fib* Fibrinogen, *D-D* D-dimer.

Results from Spearman’s correlation analysis for TGA parameters at 5 pM TF, demonstrated associations with markers of cell lysis (LDH), inflammation (CRP, IL-6), and coagulation (fibrinogen and D-dimer): (1) the lag-time was positively associated with all the above biomarkers except for fibrinogen; (2) ETP was inversely associated with LDH and D-dimer, and positively related to fibrinogen levels, as shown in Table 5 and Supplementary Fig. S2.

### Thrombin generation and clinical severity

We analyzed whether in COVID-19 patients, TGA measured in PPP with 5 pM TF associated with clinical complications. Outcomes of interest included CURB-65 score (a scoring system for assessing the severity of pneumonia), ICU admission, ARDS, death, thrombosis, and the composite for adverse events that includes ARDS, thrombosis, hemorrhage or death. Of the various outcomes considered, all of them showed a positive association with ETP, and also (except for death) with lag-time (Table 6).

**Table 6:** Association between clinical outcomes and baseline thrombin generation parameters obtained with tissue factor (5 pM) in samples from hospitalized COVID-19 patients.

	CURB-65		ICU		ARDS		Death		Thrombosis		Complication*	
	0–1 (N = 88)	≥ 2 (N = 39)	No (N = 94)	Yes (N = 33)	No (N = 88)	Yes (N = 39)	No (N = 117)	Yes (N = 10)	No (N = 120)	Yes (N = 7)	No (N = 86)	Yes (N = 41)
Lag-time	3.8 ± 0.9	4.5 ± 1.4	3.9 ± 1.1	4.4 ± 1.2	3.9 ± 1.0	4.4 ± 1.3	4.0 ± 1.2	4.1 ± 0.9	3.9 ± 1.0	5.8 ± 2.3	3.8 ± 0.9	4.5 ± 1.4
*p*	**0.004**		**0.020**		**0.018**		0.641		**0.009**		**0.020**	
ETP	1340 ± 261	1158 ± 341	1352 ± 284	1092 ± 255	1332 ± 290	1178 ± 293	1302 ± 297	1082 ± 251	1312 ± 272	808 ± 355	1382 ± 258	1080 ± 277
*p*	**0.003**		** < 0.001**		**0.008**		**0.011**		**0.001**		**0.001**	

Bold values denote statistical significance at the *p* < 0.05 level.

*ARDS* acute respiratory distress syndrome.

*Complications include ARDS, Thrombosis, Hemorrhage, or Death. *P* values were based on Mann–Whitney-U test.

**Figure 1:**
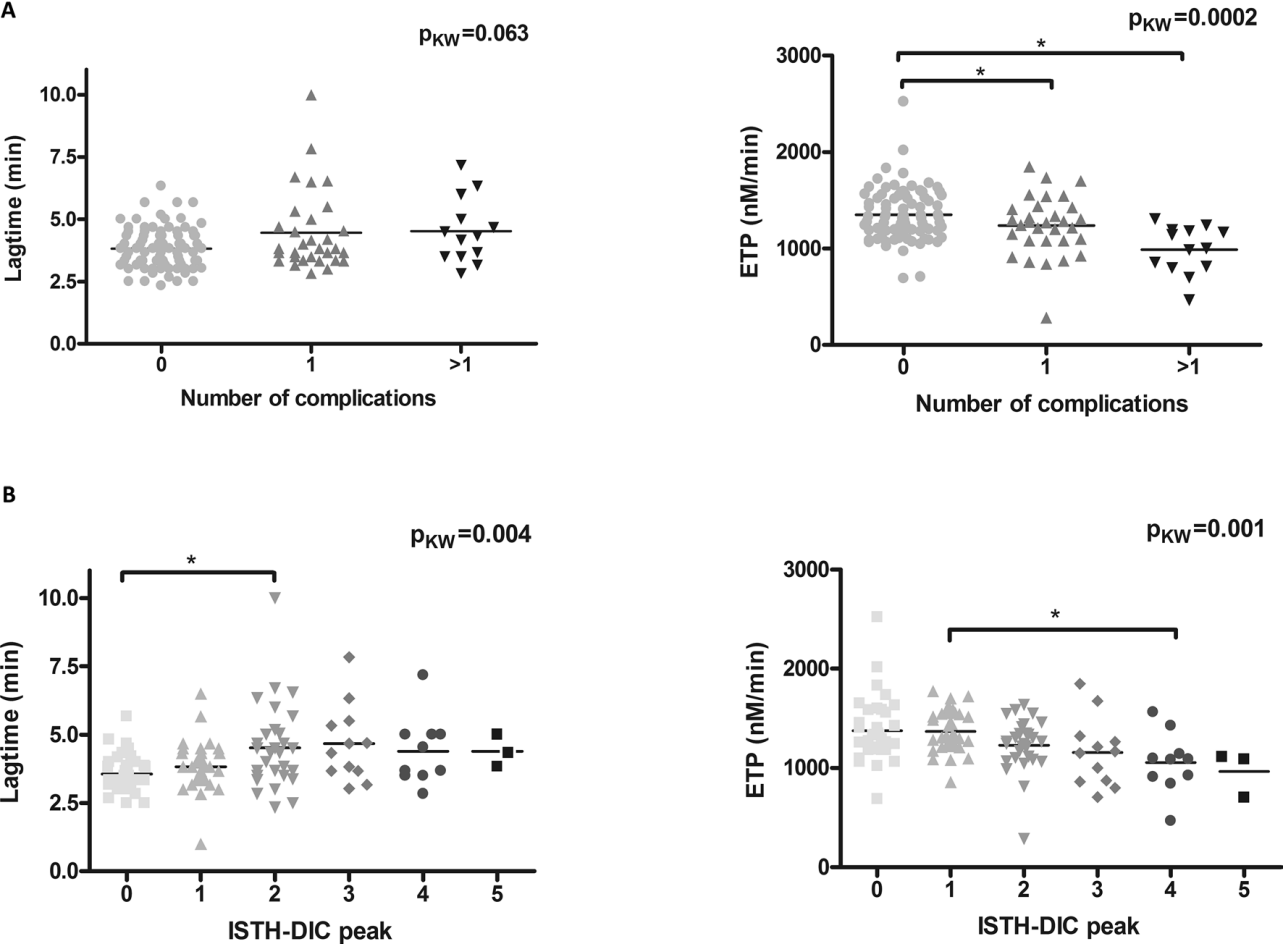
Association of lag-time and ETP values of COVID-19 patients with the number of complications (**A**) and peak ISTH-DIC scores (**B**). p_KW_: *p* value based on Kruskal Wallis test comparing all groups; *p < 0.05 based on Mann–Whitney-U test comparing pairs.

As an additional main outcome, and according to the criteria defined by the ISTH to be applied to patients with a critical illness known to precipitate DIC, the ISTH-DIC score was evaluated, and the number of clinically relevant episodes (ADRS, thrombosis, bleeding or death) were also scored during follow-up. TGA showed lower ETP levels but longer lag-times in patients who experienced more clinical events and had higher ISTH-DIC scores (Fig. 1) (Table 6).

### Predictive value of thrombin generation assay in COVID-19

**Figure 2:**
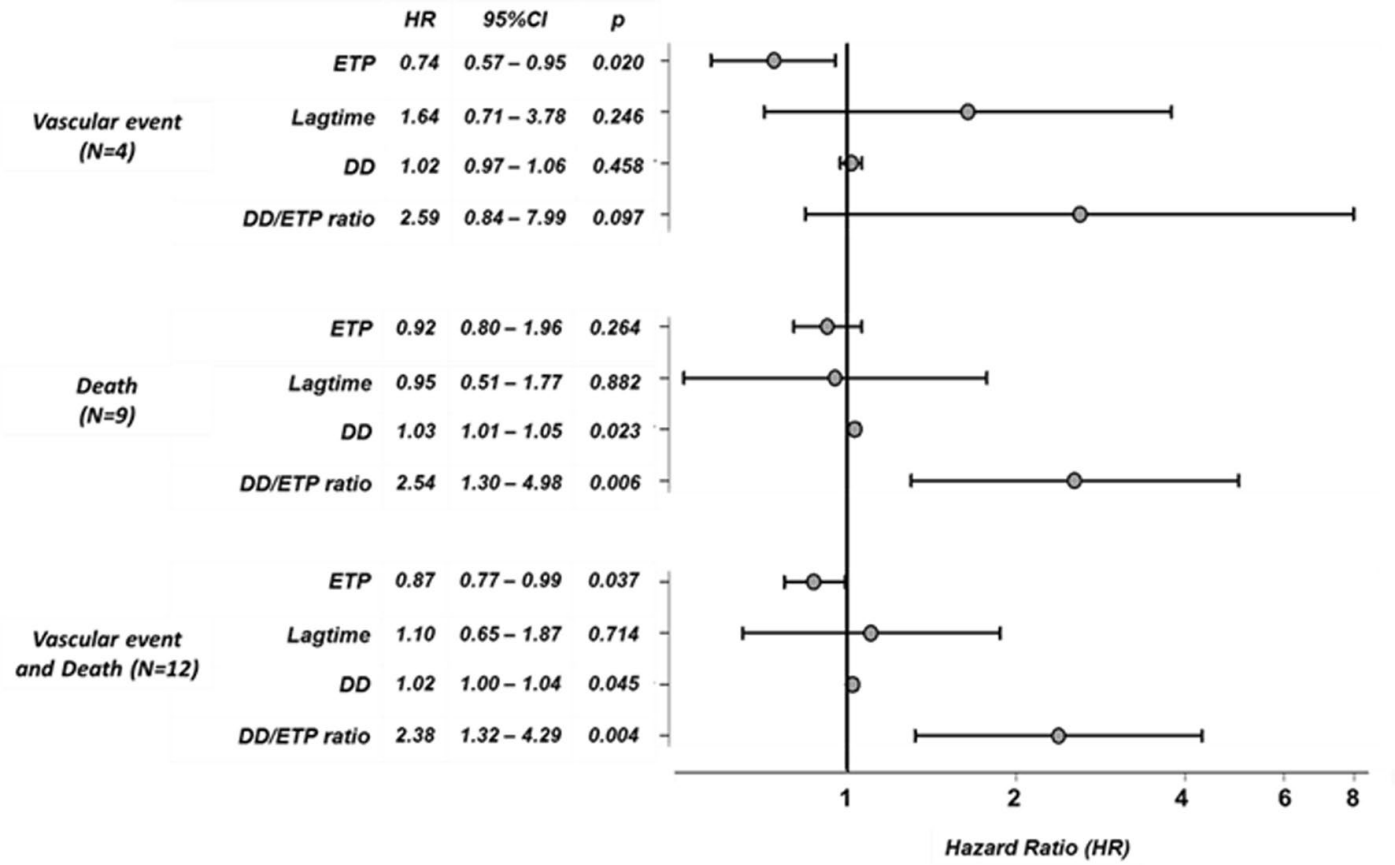
Cox regression analysis of baseline thrombin generation parameters, baseline D-dimer and the baseline D-dimer/ETP ratio in COVID-19 patients. Square root transformation of ETP and D-dimer and logarithmic transformation of D-dimer/ETP ratio were performed for predictive analysis. 95% confidence interval and *p* value are shown for each Hazard Ratio. DD: D-dimer. HR Hazard ratio.

Cox regression analysis was performed to evaluate the predictive value for baseline TGA parameters of adverse clinical events in patients hospitalized with COVID-19 infection. For this analysis, individuals experiencing adverse events within three days following sample collection were excluded (N = 6). Cox regression analysis confirmed that in spite of the low number of events, baseline ETP and baseline D-dimer significantly predicted adverse outcomes (thrombosis, bleeding or death), being the baseline D-dimer/ETP ratio the best predictor (Fig. 2).

**Figure 3:**
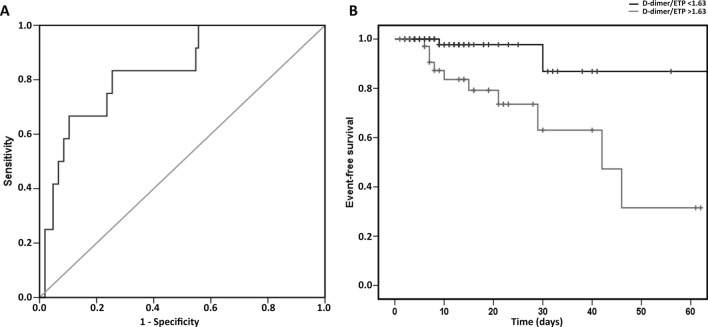
Ratio D-dimer/ETP as predictor for adverse events (vascular events or death) in COVID-19 patients. (**A**) ROC curve; (**B**) Kaplan–Meier curve.

The AUC of the ROC curve of the baseline D-dimer/ETP ratio for adverse events in COVID-19 patients was 0.833 (95%CI 0.719–0.948, *p* < 0.001) (Fig. 3A). The ROC curve showed that the cut-off value of 1.63 had a sensitivity of 83% and specificity of 75% for predicting adverse events. Using this optimal cut-off value, the Kaplan–Meier estimation also reflected that the baseline D-dimer/ETP ratio was significantly related to adverse events (*p* = 0.016, HR 0.153) (Fig. 3B).

## Discussion

It is now accepted that coagulopathy and DIC associate with a poor prognosis in patients with COVID-19, although it is as yet unknown if this is directly related to SARS-CoV-2 damage, or more likely due to the effects of hypoxia and sepsis^27^. The high incidence of thrombosis in COVID-19 strongly supports a significant disturbance of the hemostatic system in these patients, that however is only mainly sustained by the increase of D-dimer^28^. Thus, further clues on the status of the hemostatic system in patients with COVID-19 are required. Moreover, as subsequent waves of infections appear, we need new and stronger prognostic biomarkers to guide clinical decision making in order to triage which patients can be discharged or may need admission, or to identify high-risk cases that might need admission in ICU. At the present time, current prophylaxis seems to be inefficient in some patients, which could however associate with an increased bleeding risk^29^, so that more individualized approaches to guide thrombosis prevention could help in the tailored management of patients^30^. Routine coagulation tests do not seem useful to discriminate severe cases of COVID‐19, as recently suggested^19^. Clotting times (PT and aPTT) do not enable the correct appreciation of coagulation profile in patients with coagulopathy because they take into account only extreme reductions in the levels of pro‐coagulant factors and are not sensitive to the concomitant reduction of inhibitors. In this framework, global tests of hemostasis, which are closer to the in vivo coagulation conditions and reflect the capacity of response of the hemostatic system to a triggering factor, might be of great value^31^. However, to our knowledge only three studies have used other global hemostatic tests apart from coagulation times to evaluate the hemostatic system in patients with COVID-19. Such studies, two using thromboelastography and the third also using a TGA, included a relatively limited number of patients (22, 24 and 78, respectively), and the first was restricted to patients admitted to ICU^32–34^.

Here, we used a CAT TGA to analyze the response of the hemostatic system of baseline samples to TF both in the absence and presence of sTM (therefore evaluating the extrinsic pathway and the protein C system, respectively), but also by activating the contact pathway with silica. Additionally, we studied the association of baseline TGA parameters with analytical and clinical data, which may help to understand the relationship of changes in the hemostatic system with thromboinflammatory biomarkers and with clinical outcomes. For that, the assay was done in a single equipment under strict conditions aiming to reduce any variability, and with appropriate controls, which also included patients with SARS-CoV-2-negative pneumonia.

Results confirm previous findings showing a hypercoagulable state in COVID-19 patients, as they have similar thrombin generation than healthy controls despite thromboprophylaxis^32–34^. Results also reflect the impairment of the protein C pathway, as the addition of sTM, both in COVID-19, and in patients with SARS-CoV-2-negative pneumonia, resulted in minor reduction in the generation of thrombin compared to controls. These results are in line with those suggesting that infections may trigger the release of TM from the damaged endothelium^35^. This reduction of the antithrombotic capacity at the surface of the endothelium^36, 37^, may contribute to fibrin deposition, particularly at the lung^38^. Further studies of sTM in these patients might be useful to validate this hypothesis.

Our study also shows a good correlation of baseline TGA parameters with peak values of markers of cell lysis (LDH), inflammation (IL-6; CRP) and coagulation (fibrinogen and D-dimer). Interestingly, patients with higher inflammation and cell lysis have prolonged lag-time and ¨_reduced ETP, probably reflecting a consumption of coagulation factors of the hemostatic system. In this framework, the association of lag-time and ETP with D-dimer is also remarkable. The fact that patients with higher D-dimer had prolonged lag-time and reduced ETP supports a coagulopathy in severe COVID-19 patients. This finding was also reinforced by the excellent association of ETP with the ISTH-DIC score. Thus, our results would indicate both, a hypercoagulable state, and a low-grade coagulopathy in COVID-19 patients.

Finally, the association of baseline TGA parameters with clinical outcomes is also noteworthy. In contrast to that expected for a hypercoagulable state, lower ETP was associated to worse prognosis according to at-admission CURB-65 score and the development of adverse events (ARDS, vascular events, or death). Those patients with reduced capacity to generate thrombin have poor prognosis, suggesting that the severity of the disease is related to a low capacity of response of the hemostatic system, probably because of the associated consumptive coagulopathy. In accordance, our study confirms the prognostic value of D-dimer levels on mortality, which has been debated^39^. Additionally, these data also suggest that baseline TGA parameters, especially the baseline D-dimer/ETP ratio might have better prediction ability for vascular events and death. One of the strengths of the approach is the use of a representative patient sample that includes patients managed in two distinct institutions. But our study also has some limitations. First, we have no data about thrombin activity before initiation of heparin, which would allow more complete characterization of the effect of the anticoagulant on thrombin generation over time. Second, inherent to a retrospective analysis, there is a possibility that medical records were incomplete or missing data. Third, it would be interesting to evaluate whether factors such as age or sex, not matched between patients and controls in our study might play any role. Last, we did not perform a longitudinal follow-up analysis, and could not test dynamic changes of thrombin generation parameters. The variations over time of other coagulation measurements, such as prothrombin time activity^40^ and D-Dimer^41^, have already been shown to have prognostic value in patients hospitalized for COVID-19. Notwithstanding these limitations, our results suggest that thrombin generation could have a promising prognostic value in COVID-19 patients, but more extensive testing is warranted to validate these findings before being incorporated into clinical practice.

**Figure 4:**
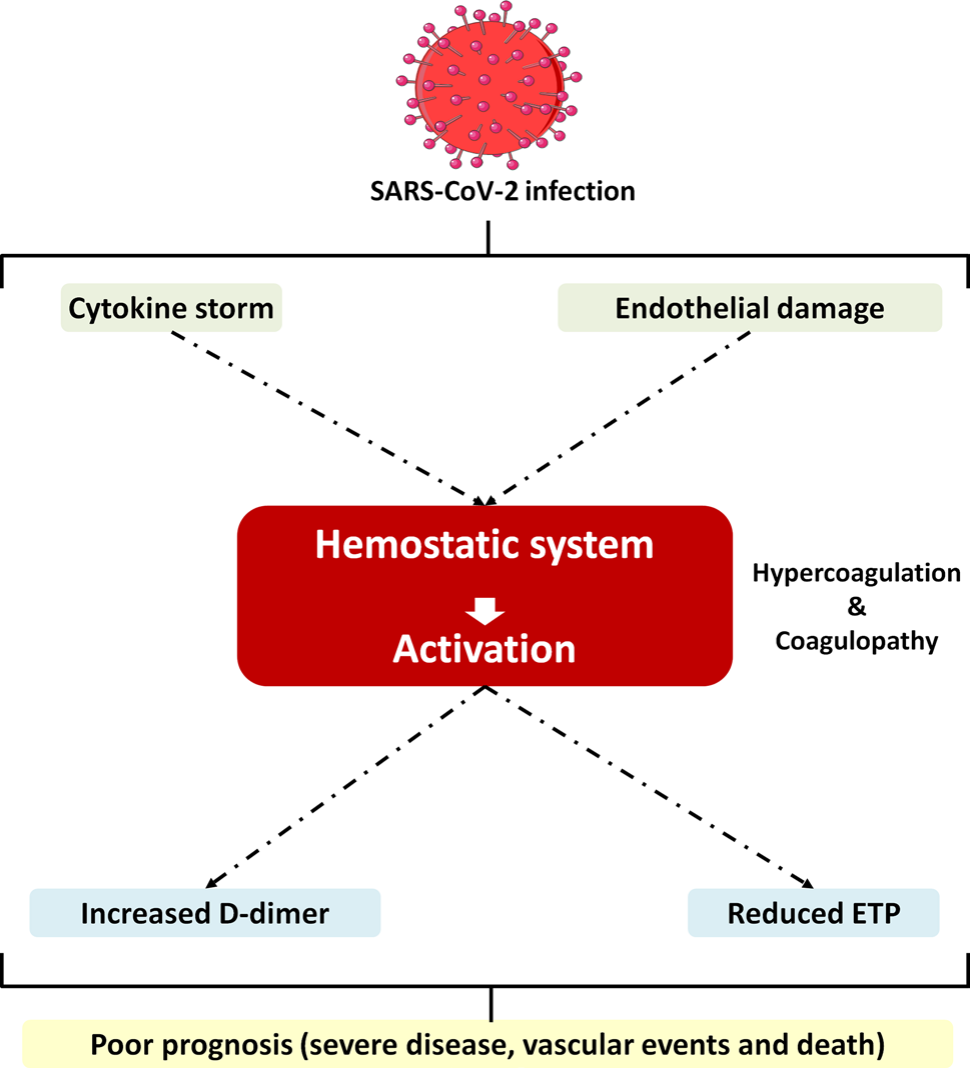
Schematic representation of potential insults associated to SARS-CoV-2 infection and the consequences on the hemostatic system. The drawing is a modified version of an image available in Servier Medical Art templates, which are licensed under a Creative Commons Attribution 3.0 Unported License; https://smart.servier.com.

Overall, our study supports the results of previous reports showing multiple pathogenic pathways in COVID-19 patients. The severe “storm” of proinflammatory cytokines, combined with cell lysis, particularly at the endothelium, constitute insults leading to a significant hypercoagulable state that, despite antithrombotic prophylaxis, cause a consumptive and diffuse coagulopathy reflected by the increase of D-dimer, independently of the hypofibrinolysis that is also present in these patients^42^. Thus, patients with lower capacity of thrombin generation and higher D-dimer levels would have poor prognosis (Fig. 4).

## Supplementary Information

Supplementary Information 1.

Supplementary Information 2.

## Data Availability

The datasets used and/or analysed during the current study are available from the corresponding author on reasonable request.
