# Supplementary figures and images for: Prognostic value of thrombin generation parameters in hospitalized COVID-19 patients
Source: Sci Rep. 2021 Apr 8;11:7792. doi: 10.1038/s41598-021-85906-y (PMC8032761; doi:10.1038/s41598-021-85906-y)

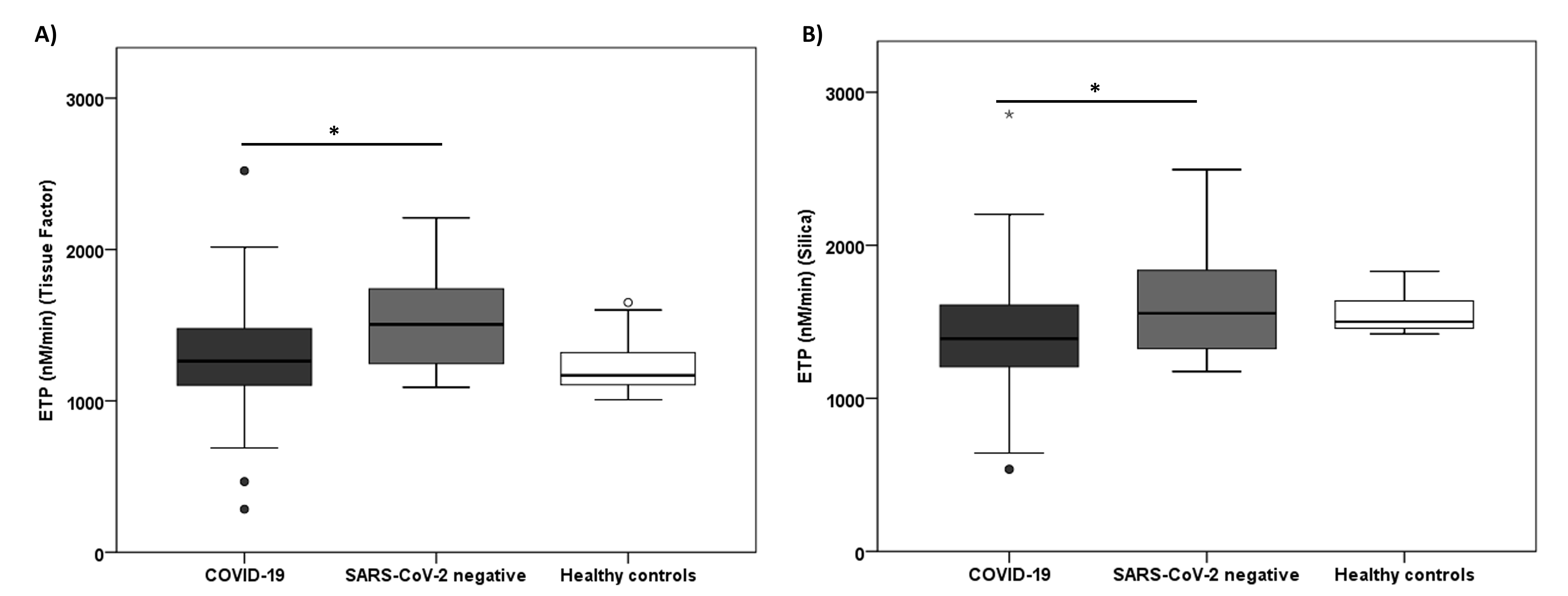

Supplement: Supplementary file 1 — Supplementary Information 1. [file 41598_2021_85906_MOESM1_ESM.tif]
